# Correlations between Limbic White Matter and Cognitive Function in Temporal-Lobe Epilepsy, Preliminary Findings

**DOI:** 10.3389/fnagi.2014.00142

**Published:** 2014-06-30

**Authors:** Ryan P. D. Alexander, Luis Concha, Thomas J. Snyder, Christian Beaulieu, Donald William Gross

**Affiliations:** ^1^Division of Neurology, University of Alberta, Edmonton, AB, Canada; ^2^Department of Biomedical Engineering, University of Alberta, Edmonton, AB, Canada; ^3^Department of Psychiatry, University of Alberta, Edmonton, AB, Canada

**Keywords:** mesial temporal sclerosis, temporal-lobe epilepsy, neuropsychological assessment, diffusion tensor imaging, processing speed

## Abstract

The limbic system is presumed to have a central role in cognitive performance, in particular memory. The purpose of this study was to investigate the relationship between limbic white matter microstructure and neuropsychological function in temporal-lobe epilepsy (TLE) patients using diffusion tensor imaging (DTI). Twenty-one adult TLE patients, including 7 non-lesional (nlTLE) and 14 with unilateral mesial temporal sclerosis (uTLE), were studied with both DTI and hippocampal T2 relaxometry. Correlations were performed between fractional anisotropy (FA) of the bilateral fornix and cingulum, hippocampal T2, neuropsychological tests. Positive correlations were observed in the whole group for the left fornix and processing speed index. In contrast, memory tests did not show significant correlations with DTI findings. Subgroup analysis demonstrated an association between the left fornix and processing speed in nlTLE but not uTLE. No correlations were observed between hippocampal T2 and test scores in either the TLE group as a whole or after subgroup analysis. Our findings suggest that integrity of the left fornix specifically is an important anatomical correlate of cognitive function in TLE patients, in particular patients with nlTLE.

## Introduction

Temporal-lobe epilepsy (TLE) is the most common focal epilepsy syndrome and is often associated with cognitive comorbidity in particular for patients with mesial temporal sclerosis (MTS) (Hermann et al., 2009). Previous magnetic resonance imaging (MRI) volumetric studies have demonstrated that cognitive deficits in TLE are most strongly predicted by white matter volume (Hermann et al., 2002, 2003). Diffusion tensor imaging (DTI) permits the measurement of specific white matter tissue characteristics and thus has potential advantages over a non-specific technique like volumetric MRI (Basser et al., 1994). *In vivo* DTI of the fornix in TLE patients has shown histological correlates between fractional anisotropy (FA) and axonal membranes (Concha et al., 2010) thereby validating the technique as a non-invasive indicator of white matter micro-structural characteristics in human brain. Diffusion parameters of the uncinate fasciculus, inferior fronto-occipital fasciculus, arcuate fasciculus, and cingulum have been demonstrated to correlate with verbal memory, naming performance, and fluency in TLE patients (Flugel et al., 2006; Diehl et al., 2008; McDonald et al., 2008; Riley et al., 2010). In the present study, we focused on the fornix and cingulum, two prominent structures in the limbic white matter network, which have been demonstrated to be abnormal in TLE, albeit with differences between non-lesional TLE (nlTLE) and TLE with unilateral MTS (uTLE) (uTLE subjects have been demonstrated to have reduced FA of the fornix and cingulum while nlTLE have not) (Concha et al., 2005a,b, 2009). The purpose of this study was to assess correlations between white matter structure using DTI and cognitive function in a group of adult TLE patients.

## Materials and Methods

### Standard protocol approvals, registrations, and patient consents

Approval of the project was obtained from the University of Alberta Health Research Ethics Board and informed consent was obtained from all participants.

### Subjects

Twenty-one left hemisphere language dominant TLE patients were studied, including 7 nlTLE patients (3 left, 1 right, 3 bilateral) and 14 with uTLE (10 left, 4 right) (Table 1). No significant differences were observed between nlTLE and uTLE group means of age (nlTLE: 42, uTLE: 41, *p* = 0.86), onset age (nlTLE: 22, uTLE: 13, *p* = 0.10), or disease duration (nlTLE: 20, uTLE: 27, *p* = 0.21). The median number of prescribed anti-epileptic drugs per patient was two in both groups. Of note, a similar number of patients in each group were prescribed topiramate (nlTLE: 3, uTLE: 4), which has been shown to negatively impact cognitive ability, particularly verbal memory, and fluency (Thompson et al., 2000). TLE diagnosis was based on ictal semiology of complex partial seizures, confined temporal-lobe interictal, and ictal electroencephalogram findings. Absence of structural lesions outside of the temporal-lobe was confirmed on clinical MRI. MTS was defined based on hippocampal T2 greater than two standard deviations of controls. Patients were considered non-lesional if hippocampal T2 was within two standard deviations of control values. Left hemisphere language dominance was confirmed by either dichotic listening or intracarotid sodium amytal tests. The patient group used in this analysis is a subset of the study group previously reported (Concha et al., 2009) exclusions were made for: insufficient neuropsychological test data, neuropsychological testing greater than 36 months from the time of research MRI, or atypical language dominance. The median length of time between research MRI and neuropsychological evaluation was 1 month (range 0–35 months), 14 patients having less than 6 months between MRI and neuropsychological evaluation. Additional patient information has been previously reported (Concha et al., 2009).

**Table 1:** **Demographic and clinical data for study patients**.

Variable		nlTLE	uTLE
Epileptic focus	Left	3	10
	Right	1	4
	Bilateral	3	–
Sex	Male	3	4
	Female	4	10
Age (years)	Mean ± SD	42 ± 10	41 ± 10
	Range	30–59	20–59
Education (years)	Mean ± SD	12 ± 2	10 ± 3
	Range	7–14	4–15
Onset age (years)	Mean ± SD	22 ± 10	13 ± 11
	Range	11–37	1–33
Disease duration (years)	Mean ± SD	20 ± 13	27 ± 13
	Range	8–47	6–51
Processing Speed Index	Mean ± SD	88 ± 15	76 ± 14
	Range	70–112	55–105
Auditory Verbal Learning	Mean ± SD	34 ± 12	29 ± 10
Test score	Range	17–53	9–50
Continuous Visual Memory	Mean ± SD	28 ± 12	23 ± 20
Test score	Range	6–42	−11–63

*No significant differences were observed between nlTLE and uTLE groups for age (*p* = 0.83), education (*p* = 0.13) onset age (*p* = 0.09), and disease duration (*p* = 0.26)*.

### Neuropsychological evaluation

The neuropsychological tests used were: Processing Speed Index (Digit Symbol and Symbol Search subtests of the WAIS-III) (*n* = 19), that has been reported to be sensitive to white matter abnormalities (Axelrod et al., 2001; Drew et al., 2009), the Rey Auditory Verbal Learning Test delayed recall score (AVLT) (*n* = 21) as a measure of verbal memory, and the Continuous Visual Memory Test total raw score (CVMT) as a measure of figural memory (*n* = 21). Standardized scores adjusted for both age and education were used.

### Image acquisition

Cerebrospinal fluid-suppressed diffusion tensor images were acquired in 9:30 min using a 1.5 T Siemens Sonata MRI scanner (Siemens Medical Systems, Erlangen, Germany). The sequence consisted of 26 contiguous 2 mm thick axial slices positioned to provide coverage of the limbic tracts with an in-plane resolution of 2 mm × 2 mm (interpolated to 1 mm × 1 mm × 2 mm). Diffusion-sensitized images were acquired in six directions, with *a b* value of 1000 s/mm^2^. Full details of the DTI protocol have been previously provided (Concha et al., 2005a,b). T2 images for the quantification of hippocampal T2 were obtained using a modified CPMG sequence with 32 echoes (TR = 4.43 s; TE_1_ = 9.1 ms, echo spacing = 9.1 ms), producing 10 coronal 3 mm thick slices with a 3 mm interslice gap in 8:13 min (voxel size 1.2 mm × 1.2 mm× 3 mm interpolated to 0.6 mm × 0.6 mm × 3 mm).

### Image processing

Bilateral fornix and cingulum were investigated using deterministic tractography. By transferring DTI images to a personal computer running DTIstudio 2.5 (Johns Hopkins University, Baltimore, MD, USA), tracts were depicted using fiber assignment by continuous tracking algorithm. Placement of tract-selection regions of interest (ROI) for each fiber bundle was based on methods outlined previously (Mori et al., 1999; Wakana et al., 2004). The FA threshold was set at 0.3 for all tracts. The FA threshold of 0.3 is commonly used in the deterministic tractography literature and is the threshold that we have used in our previous tractography studies of the fornix and cingulum (Mori et al., 1999; Concha et al., 2005a,b, 2009). If more than one streamline for a tract passed through a voxel, repeated coordinates were discarded and the voxel was only included once in the analysis. Mean diffusion parameters for each white matter tract were calculated in each patient. For this study, the crus of the fornix and the temporal portion of the cingulum were analyzed between the axial levels of the mammillary bodies and the fusion of the crura of the fornices. The T2 signal decay was fitted to a mono-exponential curve by voxel across the multi-echo coronal images. T2 values for each hippocampus were calculated by averaging within ROIs manually drawn on two consecutive slices (Concha et al., 2005a,b).

### Statistical analysis

#### Statistical tests

Analyses were performed using SPSS 17.0 (SPSS Inc., Chicago, IL, USA). Spearman correlations were used to evaluate the relationship between FA of each tract and neuropsychological test scores. With a total of 12 tests performed for the primary analysis, Bonferroni correction sets a significance level of *p* < 0.0042.

#### Primary analysis

The primary analysis was made studying the entire group (nlTLE, uTLE, right, left, and bilateral TLE combined) based on the hypothesis that correlations would be observed between white matter measures and neuropsychological tests and that the previously reported differences in white matter DTI measures and neuropsychological tests seen in nlTLE and uTLE and right and left TLE would provide an adequate range of values to demonstrate significant correlations.

#### Secondary analysis

Where significant correlations were observed in the preliminary analysis, subsequent analysis was performed looking at the nlTLE and uTLE groups and right and left TLE groups separately. Correlations between hippocampal T2 and neuropsychological test scores were also assessed based on the hypothesis that changes in either white matter diffusion parameters or cognitive test scores could be secondary to mesial temporal pathology.

## Results

### Patient neuropsychological test scores

After examining whole group summary statistics of neuropsychological test scores [Processing Speed: 80 ± 15 (55–112); AVLT: 32 ± 12 (9–57); CVMT: 23 ± 17 (−11–63)], differences between nlTLE and uTLE subgroups were also investigated (Table 1). When compared to the average processing speed standard score of 100 (SD 15), the nlTLE group mean of 88 is low average but still within normal limits, whereas the uTLE group score of 76 is mildly deficient. Considering AVLT, for which the mean standard score is 50 (SD 10), both groups are within normal limits for AVLT but nlTLE patients scored higher (54) than uTLE (42). Both groups were impaired (<first percentile) on the CVMT (nlTLE: 24, uTLE: 23) (Table 1).

### Primary analysis (Entire TLE group)

Higher FA values for the left fornix were associated with higher Processing Speed indices [*r*(*p*)′ = 0.62, *p* = 0.004] (Table 2). No other correlations were observed between Processing Speed, AVLT, CVMT, and FA of the fornix and cingulum (Table 2).

**Table 2:** **Spearman rho correlations of neuropsychological test scores and fractional anisotropy (FA) of the fornix and cingulum in TLE patients**.

	Processing Speed Index (PS)	Auditory Verbal Learning Test (AVLT)	Continuous Visual Memory Test (CVMT)
	
	*n* = 19	*n* = 21	*n* = 21
Left fornix	0.62 (0.004)*	-0.10 (0.67)	0.33 (0.15)
Right fornix	0.25 (0.30)	-0.04 (0.87)	0.27 (0.25)
Left cingulum	0.32 (0.18)	-0.09 (0.71)	-0.02 (0.92)
Right cingulum	0.26 (0.28)	0.01 (0.97)	-0.05 (0.82)

Displayed as ‘Spearmans’s *r* (*p*)’. *denotes significance with Bonferoni correction (p < 0.0042).

### Secondary analysis

#### nlTLE and uTLE groups

**Figure 1:**
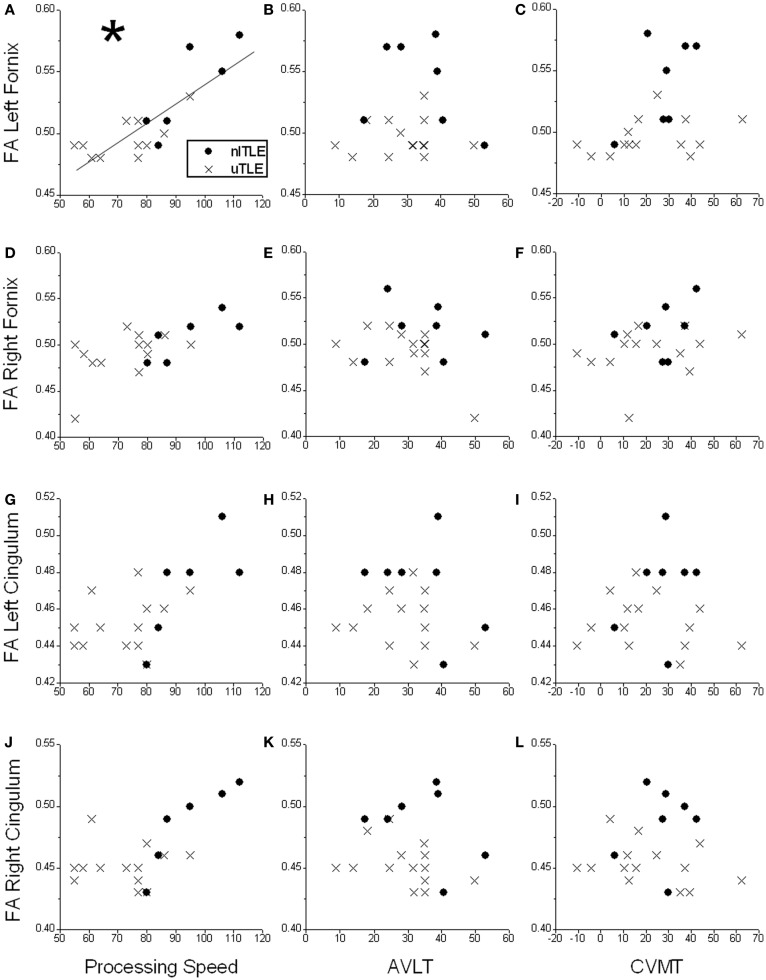
**Scatter plots displaying correlations of neuropsychological test scores [Processing Speed Index, Auditory Verbal Learning Test (AVLT), and Continuous Visual Memory Test (CVMT)] and fractional anisotropy (FA) of the left fornix (A–C), right fornix (D–F), left cingulum (G–I), and right cingulum (J–L) in patients with temporal-lobe epilepsy without mesial temporal sclerosis (nlTLE) and with mesial temporal sclerosis (uTLE)**. *A significant positive correlation is seen between Processing Speed and FA of the left fornix which is primarily driven by the nlTLE subjects. No other significant correlations were observed for the other comparisons.

After splitting the TLE patients into subgroups (nlTLE *n* = 7, uTLE *n* = 14), other trends became evident. The correlation between FA of the left fornix and Processing Speed was explained by a strong correlation in the nlTLE group [*r*(*p*)′ = 0.90, *p* = 0.02], whereas the uTLE group showed no correlation [*r*(*p*)′ = 0.40, *p* = 0.18] (Figure 1).

#### Right and left TLE

Analysis of right TLE (*n* = 5) and left TLE (*n* = 13) demonstrated a positive correlation between FA of the left fornix and processing speed in left TLE [*r*(*p*)′ = 0.60, *p* = 0.04].

#### Correlations between hippocampal T2 relaxometry and neuropsychology

Correlations were not observed between any of the neuropsychological test scores and left or right hippocampal T2 in the total patient group. As well, no correlation was seen between left hippocampal T2 and processing speed in the nlTLE group.

## Discussion

Quantitative MRI studies have shown white matter volume correlates with verbal and figural memory (Hermann et al., 2002, 2003). Recent DTI studies have demonstrated reduced FA in cerebral white matter in multiple brain regions and specific white matter tracts (Concha et al., 2005a,b, 2009; Gross et al., 2006; Focke et al., 2008; Schoene-Bake et al., 2009) with several reports demonstrating correlations between memory and DTI changes in TLE (Diehl et al., 2008; McDonald et al., 2008; Riley et al., 2010). While memory deficits are considered central to the clinical phenotype of TLE, broader cognitive measures such as intelligence, executive function and motor speed have also been demonstrated to be reduced (Hermann et al., 1997; Oyegbile et al., 2004).

The goal of this study was to investigate the relationship between limbic white matter microstructure and cognition in TLE. The primary observation of this study was that correlations were only observed between FA of the left fornix and processing speed for the TLE group as a whole with no correlations observed for the verbal (AVLT) and non-verbal (CVMT) memory tests nor for FA of the other tracts (right fornix and bilateral cingulum) and any of the cognitive measures.

It was observed that the Processing Speed Index, a WAIS-III measure that is most sensitive to brain disorders affecting white matter (Hawkins, 1998) such as traumatic brain injury (Axelrod et al., 2001) and multiple sclerosis (Drew et al., 2009), only correlated with left fornix FA whereas it is usually considered a measure of more widespread white matter function. Wernicke–Korsakoff syndrome, largely based in mammillary body pathology (Zubaran et al., 1997), has also been linked to deficits in one of the two subtests making up the Processing Speed Index, the Digit Symbol-Coding test, in addition to the expected deficit in memory test scores (Jacobson et al., 1990; Oscar-Berman et al., 2004). While there are no other specific references to the relationship between the left fornix specifically and Processing Speed, the link between Processing Speed and limbic structures both for Wernicke–Korsakoff as well as this study suggests an important role of the limbic system specifically in what has been considered a measure of more widespread white matter functioning. While the absence of correlations with other white matter structures suggests a unique role for the left fornix, given the limited sample size, false negative results cannot be ruled out, therefore, further work is required to better understand the relationship between Processing Speed and specific white matter tracts.

While caution must be taken in drawing strong conclusions from the secondary analysis, in particular given the small sample size of some of the subgroups, several interesting findings are observed in particular when looking at the nlTLE and uTLE subgroups.

We observed that the association between left fornix FA and Processing Speed is unique to the nlTLE group despite the greater deficit in intelligence scores previously shown to be evident in uTLE patients (McMillan et al., 1987). Of note, we have previously reported correlations between FA of the fornix and disease duration in nlTLE (Concha et al., 2009). Together these observations, albeit preliminary, are consistent with fornix degeneration and progressive cognitive dysfunction being secondary to ongoing seizures in nlTLE. Of note, the absence of correlations between hippocampal T2 and cognition suggests that the observed correlations between the left fornix and Processing Speed are not a secondary effect (i.e., our findings are not consistent with the nlTLE subjects developing hippocampal degeneration which then leads to downstream degeneration of the fornix and subsequent to this reduced FA).

The dissociation of findings between subgroups suggests the potential for seizure-related white matter and cognitive deterioration being unique to the nlTLE group, supporting the distinctiveness of the nlTLE and uTLE disease states. This idea has also been suggested and supported in recent DTI research (Concha et al., 2009; Kim et al., 2010; Shon et al., 2010). The fact that no correlations were observed in uTLE patients despite greater neuropsychological impairment suggests factors other than fornix damage are responsible for cognitive deficits in this group.

There has been much investigation into the extent and severity of cognitive decline in chronic TLE. Verbal memory peaks earlier and declines faster in TLE patients, especially in left TLE (Helmstaedter and Elger, 2009). A recent prospective study has demonstrated abnormal 4 year trajectory for memory as well as executive function and motor speed in TLE which was associated with baseline MRI abnormalities, lower baseline intelligence, older age, and longer duration of epilepsy (Hermann et al., 2006). As well, general intelligence has been demonstrated to be significantly reduced in earlier onset TLE, including patients without MTS (Kaaden and Helmstaedter, 2009). The cross-sectional nature of our data makes it difficult to draw conclusions regarding whether structural (fornix) or functional (processing speed) changes are progressive. Longitudinal studies are required to evaluate the relationship between functional and structural changes seen in TLE and determine whether nlTLE and uTLE may follow different trajectories.

With respect to division of our TLE sample, an appealing avenue of analysis would be to study group differences between right and left epileptic foci, as has been previously reported (Diehl et al., 2008; McDonald et al., 2008). The primary objective of this study was to use a population of patients with TLE to look for correlations between limbic white matter structure and cognitive function. The study design intentionally included uTLE, nlTLE as well as TLE patients with both right and left epileptic foci as this design was expected to provide a range of both structural and functional abnormalities [i.e., based on the literature uTLE patients were expected to have reduced limbic white matter FA (Concha et al., 2009) and lower cognitive test scores compared to nlTLE (McMillan et al., 1987) and right and left TLE patients were expected to expand the range of scores in particular tests of verbal and figural memory] (Powell et al., 2007). While it remains interesting to look at right and left differences, unfortunately, due to limitations of sample size in particular for the right TLE group (*n* = 5), it is difficult to compare right and left TLE in this study. While the left TLE group did show a significant correlation between FA of the left fornix and processing speed, the absence of positive findings in the right TLE group is most likely explained by the low sample size.

## Conclusion

In conclusion, our findings suggest that integrity of the left fornix specifically is an important anatomical correlate of cognitive function, in particular processing speed, in TLE patients. Furthermore, the differences in correlations of limbic white matter FA versus cognitive test scores in the subgroup analysis suggest that uTLE and nlTLE are distinctly different clinical and anatomical entities.

## Conflict of Interest Statement

The authors declare that the research was conducted in the absence of any commercial or financial relationships that could be construed as a potential conflict of interest.

## Abbreviations

AVLT, Rey auditory verbal learning test recall score; CPMG, Carr Purcell Meibom Gill sequence; CVMT, continuous visual memory test total score; DTI, diffusion tensor imaging; FA, fractional anisotropy; MRI, magnetic resonance imaging; MTS, mesial temporal sclerosis; nlTLE, non-lesional temporal-lobe epilepsy; *r*(*p*)′, Spearman rho correlation.; ROI, region of interest; SD, standard deviation; TLE, temporal-lobe epilepsy; uTLE, temporal-lobe epilepsy with unilateral mesial temporal sclerosis; WAIS-III, Wechsler adult intelligence scale third edition.
